# Moderate Salinity Stress Affects Expression of Main Sugar Metabolism and Transport Genes and Soluble Carbohydrate Content in Ripe Fig Fruits (*Ficus carica* L. cv. Dottato)

**DOI:** 10.3390/plants10091861

**Published:** 2021-09-08

**Authors:** Anna Mascellani, Lucia Natali, Andrea Cavallini, Flavia Mascagni, Giovanni Caruso, Riccardo Gucci, Jaroslav Havlik, Rodolfo Bernardi

**Affiliations:** 1 Department of Agriculture, Food and Environment, University of Pisa, Via del Borghetto 80, 56124 Pisa, Italy; mascellani@af.czu.cz (A.M.); lucia.natali@unipi.it (L.N.); andrea.cavallini@unipi.it (A.C.); flavia.mascagni@unipi.it (F.M.); giovanni.caruso@unipi.it (G.C.); riccardo.gucci@unipi.it (R.G.); 2 Department of Food Science, Faculty of Agrobiology, Food and Natural Resources, Czech University of Life Sciences Prague, Kamýcká 129, 16500 Prague, Czech Republic; havlik@af.czu.cz; 3 Interdepartmental Research Center Nutrafood “Nutraceuticals and Food for Health”, University of Pisa, Via del Borghetto 80, 56124 Pisa, Italy

**Keywords:** *Ficus carica* L., salinity stress, carbohydrates metabolism, RT-qPCR, qNMR, ^1^H NMR

## Abstract

Fig trees (*Ficus carica* L.) are commonly grown in the Mediterranean area, where salinity is an increasing problem in coastal areas. Young, fruiting plants of cv. Dottato were subjected to moderate salt stress (100 mM NaCl added to irrigation water) for 48 days before fruit sampling. To clarify the effect of salinity stress, we investigated changes in the transcription of the main sugar metabolism-related genes involved in the synthesis, accumulation and transport of soluble carbohydrates in ripe fruits by quantitative real-time PCR as well as the content of soluble sugars by quantitative ^1^H nuclear magnetic resonance spectroscopy. A general increase in the transcript levels of genes involved in the transport of soluble carbohydrates was observed. *Alkaline-neutral* and *Acid Invertases* transcripts, related to the synthesis of glucose and fructose, were up-regulated in ripe fruits of NaCl-stressed plants without a change in the content of D-glucose and D-fructose. The increases in sucrose and D-sorbitol contents were likely the result of the up-regulation of the transcription of *Sucrose-Synthase-* and *Sorbitol-Dehydrogenase*-encoding genes.

## 1. Introduction

Fig trees (*Ficus carica* L., Moraceae) are widely grown in the Mediterranean area for the consumption of fresh or dry fruits. In 2019, the world production of figs was about 1.3 million tonnes, with an increasing trend over the last three years. The leading country was Turkey, with a production of 310 thousand tonnes, followed by Egypt and Morocco [1]. The consumption of fig fruits, a key component of the Mediterranean diet for millennia, is also increasing [2,3,4]. Fig fruits are a source of carbohydrates, vitamins, minerals, dietary fibres and amino acids, and in recent years, a lot of effort and economic resources have been invested to enhance fruit quality and flavour, as well as extend the storability of the highly perishable fresh fruits [5,6].

*Ficus carica* is well known for its ability to tolerate water deficit and moderate salinity [7,8,9,10] that makes this species suitable to be cultivated in semi-arid environments where the use of saline or brackish water for irrigation is quite common [11]. The cultivar ‘Dottato’ is a bifera-type fig that is widely grown in Italy. Its ‘brebas’ (the first crop) are harvested between the end of June and the beginning of July, while the syconia fruit of the main crop (‘forniti’) are harvested from early August to late September [5]. This cultivar showed moderate resilience to salinity [8,10].

In many species, salt stress alters leaf carbohydrate partitioning and concentration. ^14^CO_2_ pulse-chase experiments showed an increase in mannitol and a decrease in sucrose and glucose partitioning in the leaves of salt-stressed celery and olive plants [12,13]. Salt stress also enhances fruit soluble sugar concentrations, depending on the genotype and the magnitude of stress. Salinity has been shown to reduce fruit size in many crops [14,15,16,17]. In watermelon [15], strawberries [16] and tomato [17], salt exposure improved fruit quality by increasing dry matter, soluble solids, amino acids and soluble sugars (glucose, fructose and sucrose) concentrations. In tomato plants, salinity stress doubled starch accumulation during early developmental stages; at later stages, the complete degradation of starch to soluble sugars was responsible for the increase in sugar content in ripe red fruits [18]. The main soluble carbohydrates in fig fruits are glucose and fructose, followed by sucrose [19]. Sorbitol is present at low concentrations and, therefore, fig is considered a sorbitol-poor species [20].

Many genomic tools are available for the ‘Dottato’ cultivar, including a haplotype-phased genome sequence [21,22,23] and a leaf transcriptome [18]. Previous studies from our research group showed that the ‘Dottato’ transcriptome is very different to that of another fig cultivar, ‘Horaishi’ [24], with five hundred and thirty-four putative genes specific to the Italian cultivar [25]. Some key genes involved in sugar content variability were previously identified and their expression compared between phase II (unripe fruits) and the late part of phase III (ripe fruits) of cvs. Dottato and Brogiotto [26]. In cv. Dottato, an increased expression of a gene encoding a sucrose synthase, *SUSY1*, was shown in ripe fruits; however, another gene, *SUSY6*, showed a reduced expression. The transcripts of alkaline-neutral and acid invertases increased in the mature stages except for an *Alkaline-neutral Invertase*, *INVAND*, which decreased in ripe fruits. *Sorbitol Dehydrogenase* (*SDH*)-encoding genes were up-regulated, as well as *Hexokinase* (*HEXKIN*) and *Phosphofructokinase* (*PFK*). *Sucrose-transporter*-encoding genes, *SUCTPR* and *SUCT2IS1*, were up-regulated in ripe fruits compared to unripe ones; nonetheless, *SUCT* was down-regulated. Of the analysed mannitol and hexose transporter-encoding genes, *MANT* and *HEXT6*, transcript levels did not change during ripening [26]. On the other hand, the effect of abiotic stresses on the gene expression of fig fruits has not been studied so far.

In the present study, we investigated the effect of short-term, moderate salinity stress on the expression of the same genes as mentioned above [26], involved in the synthesis, accumulation and transport of soluble carbohydrates in ripe fig fruits. The possible effect on sugar content was investigated by quantitative ^1^H nuclear magnetic resonance (qNMR) spectroscopy.

## 2. Results

### 2.1. Salt-Induced Expression of Genes Involved in Soluble Carbohydrate Metabolism and Transport

**Figure 1:**
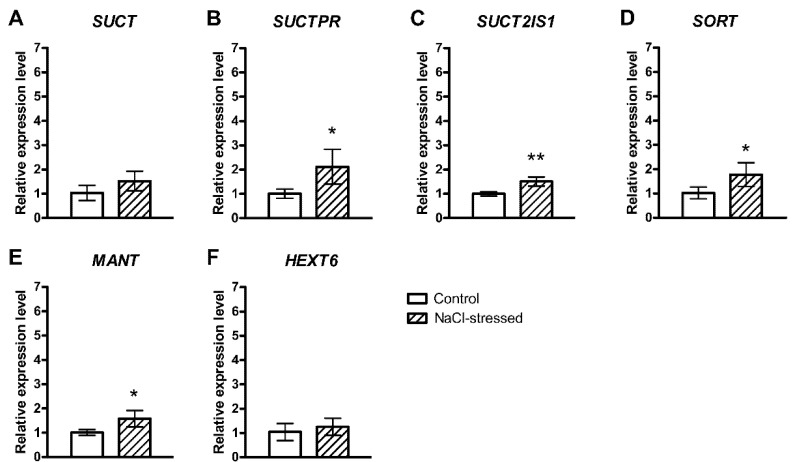
Relative expression of genes encoding carbohydrate transporters in the pulp of *F. carica* (cv. Dottato) fruits harvested from 100 mM NaCl-stressed plants after 48 days. The values were determined with RT-qPCR. (**A**) *Sucrose Transporter* (*SUCT*); (**B**) *Sucrose Transporter 4-Like* (*SUCTPR*); (**C**) *Sucrose Transporter 2 Isoform 1* (*SUCT2IS1*); (**D**) *Sorbitol Transporter* (*SORT*); (**E**) *Probable Mannitol Transporter* (*MANT*); (**F**) *Hexose Transporter 6-Like* (*HEXT6*). Fold change values are means ± SD of three biological replicates. Asterisks indicate statistically significant differences (* *p* ≤ 0.05, ** *p* ≤ 0.01).

We investigated the salinity-mediated changes in the expression of three different genes encoding sucrose transporters, including *Sucrose Transporter* (*SUCT*), *Sucrose Transporter 4 Like* (*SUCTPR*) and *Sucrose Transporter 2 Isoform 1* (*SUCT2IS1*). There were no significant differences in the expression of *SUCT* (Figure 1A), whereas *SUCTPR* and *SUCT2IS1* were up-regulated in salt-treated fruits (Figure 1B,C). Moreover, we detected the transcript levels of *Sorbitol Transporter* (*SORT*) and *Probable Mannitol Transporter* (*MANT*) genes, which were higher in the pulp of the NaCl-stressed plants than in control ones (Figure 1D,E). There were no significant differences in the expression of the *Hexose Transporter 6-Like* (*HEXT6*) gene (Figure 1F).

**Figure 2:**
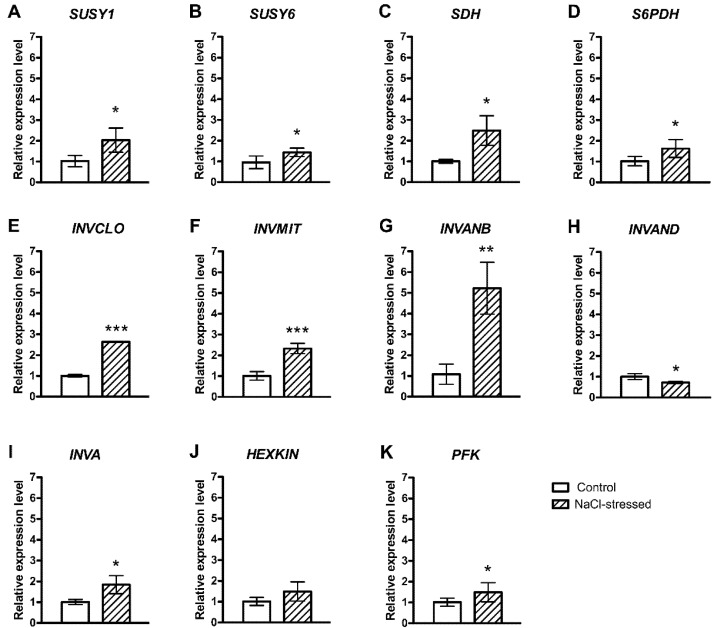
Relative expression of genes encoding carbohydrate metabolism in the pulp of *F. carica* (cv. Dottato) fruits harvested from 100 mM NaCl-stressed plants after 48 days. The values were determined with RT-qPCR. (**A**) *Sucrose Synthases 1* (*SUSY1*); (**B**) *Sucrose Synthases 6* (*SUSY6*); (**C**) *Sorbitol Dehydrogenase* (*SDH*); (**D**) *NADP-dependent D-sorbitol 6-phosphate Dehydrogenase* (*S6PDH*); (**E**) *Alkaline-neutral Invertase-Like Chloroplastic* (*INVCLO*); (**F**) *Alkaline-neutral Invertase-Like Mitochondrial* (*INVMIT*); (**G**) *Alkaline-neutral Invertase B* (*INVANB*); (**H**) *Probable Alkaline-neutral Invertase D* (*INVAND*); (**I**) *Acid β-fructofuranosidase* (*INVA*); (**J**) *Hexokinase-1* (*HEXKIN*); (**K**) *ATP-dependent-6-phosphofructokinase 3* (*PFK*). Fold change values are means ± SD of three biological replicates. Asterisks indicate statistically significant differences (* *p* ≤ 0.05, ** *p* ≤ 0.01, *** *p* ≤ 0.001).

We also analysed the expression levels of 11 key genes involved in the reversible conversion of sucrose and sorbitol into fructose and glucose. The transcript levels of *Sucrose Synthase* (*SUSY1* and *SUSY6*) genes, which catalyse the reversible conversion of sucrose into UDP-glucose and fructose [27,28], were up-regulated (Figure 2A,B). Moreover, the *Sorbitol Dehydrogenase* (*SDH*) gene, encoding the enzyme for the conversion of sorbitol into fructose [27,28,29], was up-regulated (Figure 2C) as well as the expression of the *NADP-dependent D-sorbitol 6-phosphate Dehydrogenase* gene (*S6PDH*), which is related to the conversion of glucose 6-phosphate into sorbitol 6-phosphate [27,28,29] in response to salinity (Figure 2D). All analysed invertase-encoding genes involved in the conversion of sucrose into glucose and fructose [27,28] were up-regulated, such as *Alkaline-neutral Invertase-Like Chloroplastic* (*INVCLO*), *Alkaline-neutral Invertase-Like Mitochondrial* (*INVMIT*), *Alkaline-neutral Invertase B* (*INVANB*) and *Acid β-fructofuranosidase* (*INVA*) but not *Probable Alkaline-neutral Invertase D* (*INVAND*), which was down-regulated (Figure 2E–I). The *Hexokinase-1* gene (*HEXKIN*) transcript level was unaffected by salinity (Figure 2J), whereas the second analysed kinase, the *ATP-dependent 6-phosphofructokinase 3* (*PFK*) was up-regulated in fruits of NaCl-stressed plants (Figure 2K).

### 2.2. Changes in Main Soluble Carbohydrates Contents in Response to Salinity

**Figure 3:**
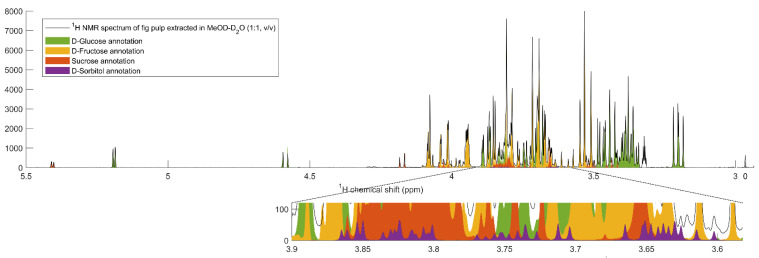
Representative annotated ^1^H NMR spectrum of fig pulp extracted in MeOD-D_2_O (1:1, *v*/*v*).

The D-glucose, D-fructose, D-sorbitol, D-mannitol and sucrose concentrations in the fruit pulp were determined by NMR quantitative analysis to investigate whether the differences in key soluble carbohydrate pathway-related genes could affect the soluble carbohydrate contents of NaCl-stressed plants. The spin systems of D-Mannitol were not unequivocally identified (Figure 3).

Total soluble carbohydrate, glucose and fructose concentrations were unaffected by salinity, but those of sucrose and D-sorbitol were higher in NaCl-stressed fruits than in control fruits (Table 1). The ratios between soluble sugars reported in Table 1 were generally higher in the stressed treatment, except for glucose/fructose. The ratios of sucrose/fructose + glucose and D-sorbitol/fructose + glucose were significantly higher in the fruits of NaCl-stressed plants than in the control fruits (Table 1). 

## 3. Discussion

Fig plants show several adaptive responses to salinity, which makes this species suitable for cultivation in moderately saline soils [8,10]. However, there is no information on the effect of salt stress on soluble sugar concentrations, a key attribute of fruit quality. Fig fruits can accumulate high amounts of soluble sugars, up to 50% of their dry weight at ripening [19]. Therefore, we investigated how salinity affected carbohydrate metabolism by comparing the transcript level of the main genes involved in the synthesis, accumulation and transport of soluble carbohydrates in ripe fruits of cv. Dottato plants grown under saline conditions.

The genes encoding sugar transporters analysed in this study showed a significant increase in the transcript levels in the fruit pulp of NaCl-stressed plants compared to controls, except *SUCT* and *HEXT6*, whose transcript level increases were not significant (Figure 1). We found that salt stress induced a general increase in the expression of genes related to carbohydrate transport, similar to results obtained in tomato fruits [18]. The expression of *MANT* was also higher in salt-stressed fruits (Figure 1E), but it has to be considered that we were unable to quantify the mannitol concentration by qNMR. This might be due to the low sensitivity of qNMR for the detection of mannitol in the dry fig matrix or to the absence of mannitol. To the best of our knowledge, mannitol has not been quantified in *Ficus* spp. tissue so far. Mannitol is a polyol that can confer resistance to oxidative stress [30,31,32] and salt tolerance [33] because it may play multiple roles as a compatible solute, a low molecular weight chaperone, a reactive oxygen species scavenging compound, an osmolyte and an osmoprotectant [34]. In our experiment, fig *MANT* transcript levels increased in the mature fruits of NaCl-stressed plants (Figure 1E), as already observed in olive fruits, in which the *OeMaT1* transcripts increased throughout salinity stress, suggesting that this gene was involved in the accumulation of mannitol for salt tolerance [35].

Among the analysed genes encoding enzymes of carbohydrate metabolism, it should be noted that the expression level of both sucrose-synthase-encoding genes increased under salinity stress, with a major expression level for *SUSY1* (Figure 2A). In NaCl-stressed tomato plants, an increased expression of a gene encoding a *Sucrose Synthase*, *SUS3*, was shown; however, another gene, *SUS2*, showed a reduced expression [36]. Salinity stress promoted sucrose translocation in the fruit [37], increasing its concentration, [17] and increased sucrose synthase activity in tomato fruits [17,37]. This is consistent with a higher concentration of sucrose in fig fruits grown under salinity conditions compared to control fruits.

The *INVAND* gene was the only gene whose transcript levels decreased in fruits of NaCl-stressed plants (Figure 2H), while the transcripts of other invertase-encoding genes increased (Figure 2E–G,I). Similar differences among invertase-encoding genes were reported in tomato fruits in response to salinity [36], where salinity increased the expression levels of the *Tiv-1* gene and reduced those of *Lin5* [36]. 

In addition, the *PFK* transcript levels increased in mature fig fruits of salt-treated plants (Figure 2K). It has been evidenced that phosphofructokinase-encoding genes play diverse functional roles in different tissues [38] including stress responses, as observed in rice seedlings [39].

In many species, salt stress affects carbohydrate contents in the fruit, depending on the genotype and the magnitude of stress. For example, a difference in the partitioning of assimilates in salinity stress conditions has been reported in tomato fruits [18,36,40,41]. Few studies have investigated changes in soluble carbohydrates in fig fruits. Despite differences among cultivars, fructose and glucose are the most abundant sugars reported in *F. carica*, followed by sucrose [26,42,43,44]. In this work, we confirm that major soluble carbohydrates in fig fruit were fructose, glucose and sucrose (Table 1). The D-glucose/D-fructose content ratio was about 1:1 and remained fairly constant under saline conditions. On the other hand, sucrose and sorbitol were significantly higher in the fruits of salt-stressed plants (Table 1), which suggests a different partitioning towards translocatable sugars in the fruit. In salt-stressed tomato fruit, the sucrose content rose, whereas the glucose and fructose contents were unaffected by salinity [17], despite glucose and fructose increasing in watermelon cultivated under salinity [15]. Nevertheless, in strawberry, glucose, fructose, sucrose and starch content reduced in all plant organs, including the fruits, due to NaCl salinity [16]. 

D-sorbitol is a well-known osmolyte that plays various roles in response to salinity stress [34]. Sorbitol has also been implicated in drought mitigation in sink organs of peach [45]. Higher concentrations of sorbitol in the fruits of plants grown in stressed conditions also suggest a possible key role for sorbitol in fig. Recent studies have reported the advantages of having sorbitol in addition to sucrose as the main translocatable sugars in apple trees to maintain the glucose and fructose levels to near homeostasis [46].

In conclusion, we showed that salinity affected the expression of main sugar metabolism and transport genes in fig fruits. A general increase in the transcript levels of genes involved in transport was observed. The increase in the transcripts encoding the enzymes involved in the synthesis of glucose and fructose did not increase the content of D-glucose and D-fructose, which are the most readily metabolised sugars. Perhaps an up-regulation of *Sorbitol Dehydrogenases* could lead to the accumulation of D-sorbitol using glucose and fructose since there was an increase in D-sorbitol.

## 4. Materials and Methods

### 4.1. Plant Material and Salt Treatment

Sixteen plants of *F. carica* cv. Dottato (five years old), propagated by rooted cuttings from the same mother plant, were trained to a single stem and grown in a glasshouse [26]. The substrate was a mixture of 6.4% clay, 8.6% silt and 85% sand. All plants were watered until saturation with tap water three times a week before we started the experiment. From the middle of June, half of the plants were irrigated three times a week with 700 mL of 50 mM NaCl solution for one week and then with the final 100 mM NaCl solution for the following 42 days (salt-treated plants) using distilled water. The step increment was used to alleviate the shock effect of salt and reach the final concentration gradually. The remaining eight control plants were similarly only irrigated with distilled water. The saline solution was obtained by adding NaCl (purity > 99.8%) (Sigma-Aldrich Co., St. Louis, MO, USA) to distilled water. Ripe fruits were sampled during the last part of phase III [47], then peeled and frozen in liquid nitrogen. The pulp (infructescence and seeds) was stored at −80 °C until analysis [26]. The sampled fruits from control and 100 mM NaCl-stressed plants were similar in morphology and colour (Figure S1, Table S1).

### 4.2. Nucleic Acid Isolation and Analysis of Gene Expression

Frozen fruit pulp was ground in liquid nitrogen and 100 mg was used for the extraction of total RNA using the RNeasy^®^ Mini Plants Kit (Qiagen, Hilden, Germany). Quantification of the total RNA samples was measured using a Qubit-iT^®^ RNA BR Assay Kit (Life Technologies, Carlsbad, CA, USA) and the integrity was evaluated by visual observation on agarose gel electrophoresis.

The RNA samples after treatment with an Amplification Grade DNase I kit (Sigma-Aldrich, Saint Louis, MO, USA) was reverse transcripted to the first-strand cDNA using a Maxima First Strand cDNA Synthesis Kit (Thermo Fisher Scientific, Waltham, MA, USA) following the manufacturer’s instructions. The calibration transcription rate of the cDNA template for the following expression analysis was established by agarose gel electrophoresis of the RT-qPCR product using the primer Universal 18S ribosomal gene (QuantumRNA, universal 18S Internal Standard; Applied Biosystems/Ambion, Foster City, CA, USA).

Analysis of gene expression was carried out by RT-qPCR using Fast SYBR^®^ Green Master Mix (Applied Biosystems, Foster City, CA, USA) with specific primers for each gene [26] in a StepOne^®^ real-time PCR System (Applied Biosystems, Foster City, CA, USA) using the thermal cycling conditions reported in the use manual. The β-tubulin gene was chosen as the housekeeping gene to normalise the relative expression of each gene for both salt-stressed and control samples [26]. The amplification of the selected genes and the reference genes were run using three biological replicates and with three technical replicates each. The relative abundance of transcripts was calculated by using the 2^−ΔΔCt^ method [48].

### 4.3. Quantitative ^1^H Nuclear Magnetic Resonance (NMR) for the Determination of Free Soluble Carbohydrates

All chemicals and reagents used were of analytical grade. Potassium dihydrogen phosphate (99%, KH_2_PO_4_), deuterium oxide (99.9%, D_2_O), methanol-d4 (>99.8%, MeOD) and methanol were purchased from VWR (Radnor, PA, USA). Sodium deuteroxide 40% *w*/*v* solution in D_2_O (99.5%, NaOD) was obtained from Alfa Aesar (Kandel, Germany). The 3-(trimethylsilyl) propionic-2,2,3,3-d4 acid sodium salt (99%, TSP), D-sorbitol (≥98%), D-fructose (≥98%), D-glucose (≥98%), sucrose (≥98%) and D-mannitol (≥98%) were purchased from Sigma-Aldrich (St. Louis, MO, USA). 

Amounts of 500 μL of MeOD and 500 μL of KH_2_PO_4_ buffer (90 mM, pH 6.0) in D_2_O containing 0.01% TSP (*w*/*v*) were added to 50 mg of the finely ground fig pulp. The mixture was vortexed at room temperature for 1 min, ultrasonicated for 15 min and centrifuged at 24,400× *g* for 10 min. An aliquot of 600 µL of the supernatant liquid was transferred to NMR tubes. The phosphate buffer was prepared by adding 90 mM of KH_2_PO_4_ and 0.01% of TSP. The pH was adjusted to 6.0 using 1.0 M NaOD [49].

All spectra were recorded at 298 K (25 °C) on a Bruker Avance III HD spectrometer equipped with a broadband fluorine observation (BBFO) SmartProbe™ with z-axis gradients (Bruker BioSpin GmbH, Rheinstetten, Germany), operating at a ^1^H NMR frequency of 500.23 MHz. The spectrometer transmitter was locked to MeOD, and all the spectra were recorded with the Bruker pulse sequence ‘noesypr1d’ for presaturation of the water signal at 4.704 ppm. Each sample was collected into 64 k data points after 128 scans and 4 dummy scans using a spectral width of 8000 Hz. The receiver gain was set to 18, the relaxation delay of 1 s, the acquisition time of 4 s and mixing time of 0.1 s. The free induction decay was multiplied by 0.3 Hz line broadening before Fourier transformation. TSP was used for calibration at 0.0 ppm.

The ^1^H NMR spectra were phased and baseline corrected using Chenomx NMR suite 8.5 software, professional edition (Chenomx Inc., Edmonton, AB, Canada). The signal assignment was performed using an in-house database and spiked samples.

### 4.4. Experimental Design and Statistical Analysis of Data

Plants were arranged in a completely randomised experimental design in a glasshouse. Three fully ripe fruits were sampled from three different plants for each treatment (control and salt-treated). The data for gene expression and sugar content were analysed by the Student’s *t*-test using GraphPad Prism version 5.00 (GraphPad software, San Diego, CA, USA). Statistical significance was considered to occur with a *p*-value ≤ 0.05. 

The statistical analysis for the RT-qPCR was performed by the authors from the Department of Agriculture, Food and Environment, University of Pisa. The ^1^H NMR analysis was performed by the Department of Food Science, Czech University of Life Sciences, Prague.

## Figures and Tables

**Table 1:** Concentrations of D-fructose, D-glucose, sucrose and D-sorbitol and their ratios in *F. carica* cv. Dottato fruits from control and 100 mM NaCl-stressed plants for 48 days. Values are means ± SD of three replicate fruits expressed as mg g^−1^ of fruit dry pulp. *p* ≤ 0.05 is in bold font. The ratio values are expressed as 100 times the real values.

Soluble Carbohydrates	Control	100 mM NaCl-Stressed	*p*-Value
D-Fructose	242.68 ± 7.73	235.87 ± 3.64	0.24
D-Glucose	240.92 ± 15.71	219.30 ± 5.29	0.09
Sucrose	27.9 ± 0.3	34.31 ± 3.51	**0.03**
D-Sorbitol	0.99 ± 0.23	1.48 ± 0.15	**0.04**
Total	511.68 ± 23.04	490.97 ± 2.14	0.18
Ratios			
Glucose/Fructose	99.22 ± 3.76	92.99 ± 3.04	0.09
Sucrose/Fructose	11.5 ± 0.25	14.55 ± 1.55	**0.03**
Sucrose/Glucose	11.61 ± 0.64	15.68 ± 1.95	**0.03**
Sucrose/Glucose + Fructose	5.78 ± 0.22	7.55 ± 0.87	**0.03**
Sorbitol/Sucrose	3.55 ± 0.82	4.35 ± 0.69	0.26
Sorbitol/Glucose	0.42 ± 0.12	0.68 ± 0.08	**0.03**
Sorbitol/Fructose	0.41 ± 0.10	0.63 ± 0.06	**0.03**
Sorbitol/Glucose + Fructose	0.21 ± 0.05	0.33 ± 0.03	**0.03**

## Data Availability

Data is contained within the article.

## Supplementary Materials

The following are available online at https://www.mdpi.com/article/10.3390/plants10091861/s1, Figure S1: Ripe fruit of *Ficus carica* cv. Dottato during the salinity experiment, Table S1: Fruit fresh weight of control and 100 mM NaCl-stressed plants for 48 days.
