# Supplementary material for: Moderate Salinity Stress Affects Expression of Main Sugar Metabolism and Transport Genes and Soluble Carbohydrate Content in Ripe Fig Fruits (Ficus carica L. cv. Dottato)
Source: Plants (Basel). 2021 Sep 8;10(9):1861. doi: 10.3390/plants10091861 (PMC8471620; doi:10.3390/plants10091861)
Supplement: Supplementary file 1 [file plants-10-01861-s001.zip › plants-1342656-supplementary.pdf]

Supplementary material

Communication

Moderate salinity stress affects expression of main sugar metabolism and transport genes and soluble carbohydrate content in ripe fig fruits (*Ficus carica* L. cv. Dottato)

Anna Mascellani, Lucia Natali, Andrea Cavallini, Flavia Mascagni, Giovanni Caruso, Riccardo Gucci, Jaroslav Havlik and Rodolfo Bernardi*

* Correspondence: rodolfo.bernardi@unipi.it

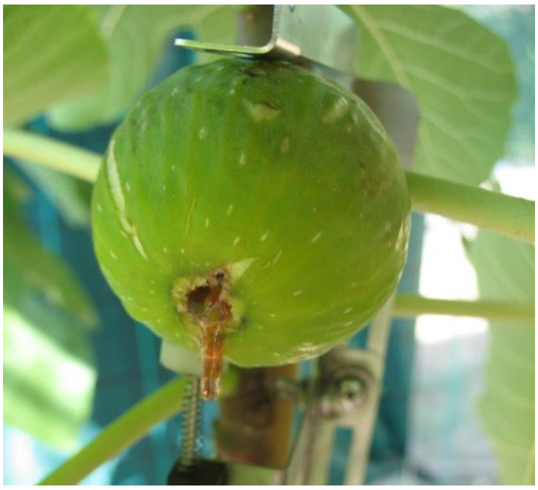

Figure S1. Ripe fruit of *Ficus carica* cv. Dottato during the salinity experiment.

Table S1. Fruit fresh weight of control and 100 mM NaCl-stressed plants for 48 days. Values are means ± standard deviations (SD) of three replicate fruits expressed as g of fresh fruit. *p*-value was calculated by Student's *t*-test.

	Control	100 mM NaCl-stressed	p-value
Replicate 1	40.56	24.75	-
Replicate 2	53.19	30.37	-
Replicate 3	29.41	23.22	-
Mean ± SD	41.05 ± 11.90	26.11 ± 3.76	0.11
